# Molecular analysis of hyperthermophilic endoglucanase Cel12B from *Thermotoga maritima* and the properties of its functional residues

**DOI:** 10.1186/1472-6807-14-8

**Published:** 2014-02-17

**Authors:** Hao Shi, Yu Zhang, Liangliang Wang, Xun Li, Wenqian Li, Xiangqian Li, Fei Wang

**Affiliations:** 1 College of Chemical Engineering, Nanjing Forestry University, Nanjing 210037, China; 2 Jiangsu Key Lab of Biomass-Based Green Fuels and Chemicals, Nanjing 210037, China; 3 Department of Life Science and Chemistry, Huaiyin Institute of Technology, Huaian 223003, China

**Keywords:** Cellulose, Conserved amino acid residues, Endoglucanase, Phylogenetic analysis, Thermostability

## Abstract

**Background:**

Although many hyperthermophilic endoglucanases have been reported from archaea and bacteria, a complete survey and classification of all sequences in these species from disparate evolutionary groups, and the relationship between their molecular structures and functions are lacking. The completion of several high-quality gene or genome sequencing projects provided us with the unique opportunity to make a complete assessment and thorough comparative analysis of the hyperthermophilic endoglucanases encoded in archaea and bacteria.

**Results:**

Structure alignment of the 19 hyperthermophilic endoglucanases from archaea and bacteria which grow above 80°C revealed that Gly30, Pro63, Pro83, Trp115, Glu131, Met133, Trp135, Trp175, Gly227 and Glu229 are conserved amino acid residues. In addition, the average percentage composition of residues cysteine and histidine of 19 endoglucanases is only 0.28 and 0.74 while it is high in thermophilic or mesophilic one. It can be inferred from the nodes that there is a close relationship among the 19 protein from hyperthermophilic bacteria and archaea based on phylogenetic analysis. Among these conserved amino acid residues, as far as Cel12B concerned, two Glu residues might be the catalytic nucleophile and proton donor, Gly30, Pro63, Pro83 and Gly227 residues might be necessary to the thermostability of protein, and Trp115, Met133, Trp135, Trp175 residues is related to the binding of substrate. Site-directed mutagenesis results reveal that Pro63 and Pro83 contribute to the thermostability of Cel12B and Met133 is confirmed to have role in enhancing the binding of substrate.

**Conclusions:**

The conserved acids have been shown great importance to maintain the structure, thermostability, as well as the similarity of the enzymatic properties of those proteins. We have made clear the function of these conserved amino acid residues in Cel12B protein, which is helpful in analyzing other undetailed molecular structure and transforming them with site directed mutagenesis, as well as providing the theoretical basis for degrading cellulose from woody and herbaceous plants.

## Background

Cellulose is the most abundant organic compound and renewable carbon resource on earth [1]. Biodegradation of cellulose, an abundant plant polysaccharide, is a complex process that requires the coordinate action of three enzymes, among which endoglucanases (EC 3.2.1.4), are able to break the internal bonds of cellulose, and disrupt its crystalline structure, exposing the individual cellulose polysaccharide chains, playing in most important role [2-4]. The degradation is mainly carried out by bacteria, fungi, and protozoa, commensals in the guts of herbivorous animals, as well as the termite *Reticulitermes speratus*[5], from which, there are variety of endoglucanases. The complex chemical nature and heterogeneity of cellulose account for the multiplicity of endoglucanases produced by microorganisms. The activity of different endoglucanases with subtle differences in substrate specificity and mode of action contributes to improvement of the degradation of plant cellulose in natural habitats. There are fourteen families of glycoside hydrolases (GHF) that are used for cellulose hydrolysis [6]. More and more extremophiles have been studied in recent years, especially the hyperthermophilic enzymes. Based on amino acid sequence homologies and hydrophobic cluster analysis, hyperthermophilic endoglucanases obtained from extremophiles, which are widely distributed in terrestrial and marine hydrothermal areas, as well as in deep subsurface oil reservoirs, have been classified into GHF12 [7-14]. As described above, there are hyperthermophilic endoglucanases from archaea, most of which were chosen for sequencing on the basis of their physiology [15]. In addition, many hyperthermophilic endoglucanases gene which have been cloned were found in some heat-tolerant bacteria [16]. Those hyperthermophilic endoglucanases have a common feature that the amino acid sequences are mostly relatively short (less than 400 amino acid residues).

Although many hyperthermophilic endoglucanases of GHF12 amino acids have been reported from archaea and bacteria, a complete survey and classification of all sequences in these species from disparate evolutionary groups, and the relationship between their molecular structures and functions are lacking. The completion of several high-quality gene or genome sequencing projects provided us with the unique opportunity to make an unprecedented assessment and thorough comparative analysis of the hyperthermophilic endoglucanases encoded in archaea and bacteria. The analysis of the full set of hyperthermophilic endoglucanases genes in genomes from diverse species allows a definitive classification of hyperthermophilic endoglucanases and an assessment of their origins, evolutionary relations, patterns of differentiation, and proliferation in the various phylogenetic groups. We are interested in finding answers to the following questions: 1) What are the evolutionary relations among these hyperthermophilic endoglucanases?; 2) What is the common feature between these conserved amino acid residues and 3D topological structure?; 3) What the mechanism of the heat tolerance among these hyperthermophilic endoglucanases?

The broad analysis in this study provided a comprehensive classification scheme and proposed a molecular structure applicable to all hyperthermophilic endoglucanases. A clear picture of the patterns of endoglucanases classes in different species groups was provided. We identified and classified in this study a higher number of hyperthermophilic endoglucanase amino acids from the GHF12 than previously reported, allowing us to identify their relationships based on the phylogenetic clustering. We found that, similar to archaea, amino acids from hyperthermophilic bacteria are also quite different from the other sequences in GHF12. We characterized several conserved amino acid sites from these endoglucanases and predicted their functionality based on the amino acids similarity among the proteins available in databases. The resulting rich data set of hyperthermophilic endoglucanases from GHF12, comprising 19 sequences, is available downloaded from NCBI (Table 1).

**Table 1:** The phylogenetic distribution of endoglucanases from glycoside hydrolase family 12

	**Organism**	**Length**	*GenBank number
Euryarchaeota	*Acidilobus saccharovorans*	396	ADL19785
	*Ignisphaera aggregans*	360	ADM27702
	*Metallosphaera cuprina*	326	AEB95090
	*Pyrococcus furiosus*	319	AAD54602
	*Sulfolobus acidocaldarius*	311	AAY81158
	*Sulfolobus islandicus*	332	ADX81754
	*Sulfolobus islandicus*	334	ACP37717
	*Sulfolobus islandicus*	334	ACR41545
	*Sulfolobus islandicus*	334	ADX84872
	*Sulfolobus solfataricus*	334	AAK42142
	*Thermococcus sp.*	319	EEB73588
	*Thermoproteus tenax*	263	CCC81038
	*Thermoproteus uzoniensis*	252	AEA12777
	*Vulcanisaeta distributa*	330	ADN509821
Bacteria	*Acidobacterium sp.*	439	ZP_07030982
	*Bacillus licheniformis*	261	AAP44491
	*Dictyoglomus turgidum*	288	YP_002352530
	*Paenibacillus mucilaginosus*	266	AEI43442
	*Spirochaeta thermophila*	438	ADN02999
	*Spirochaeta thermophila*	433	AEJ62362
	*Teredinibacter turnerae*	278	ACR14297
	*Thermobispora bispora*	393	ADG87082
	*Thermotoga naphthophila*	274	YP_003346783
	*Thermotoga maritima*	275	Z69341
	*Lysobacter enzymogenes*	383	ABI54135
	*Bacillus megaterium*	345	ADE69644
	*Streptococcus dysgalactiae*	366	BAH80742
	*Streptococcus dysgalactiae*	366	YP_002995956
	*Bacillus thuringiensis*	349	ZP_04083086
Fungi	*Stachybotrys echinata*	237	AF435067
	*Aspergillus fumigatus*	378	EDP50688
	*Aspergillus fumigatus*	378	XP_751495
	*Neosartorya fischeri*	381	XP_001266710
	*Aspergillus niger*	396	XP_001400178
	*Penicillium marneffei*	379	XP_002147625
	*Talaromyces stipitatus*	503	XP_002481822
	*Ajellomyces dermatitidis*	357	XP_002621187
Planta	*Arabidopsis thaliana*	484	BAB11001
	*Thalassiosira pseudonana*	499	XP_002287341
Insect	*Reticulitermes speratus*	448	AB019095

*All the sequences are downloaded from GenBank (http://www.ncbi.nlm.nih.gov/protein/).

## Results

### Protein sequences characteristics

GenBank has grown fast in recent years and offer us with much better taxonomic sampling for such BLAST-based analysis [17]. We performed similar BLAST-based analysis for the 19 thermophilic endoglucanase protein sequences (which included the *T. maritima* endoglucanase sequences), using the nonredundant (*nr*) database as a reference and recording highest ranking matches. We also searched endoglucanase sequences in several plants, bacteria, fungi and algae sequences including the sequences of the *R. speratus*, using the protein BLAST search engine with a variety of endoglucanase amino acid sequences as queries for most of the thermophilic endoglucanase, else using endoglucanase as a keyword for searching other amino acid sequences of endoglucanase (Table 1). In most cases, whenever significant similarity to an endoglucanase sequence was identified, the amino acid sequence was excised and homology based protein predictions were performed using the most similar query as a guide. All of these 40 protein sequences range from 252 to 438 amino acid residues in length. Of these sequences, those from archaea and bacteria showed similar lengths, especially for those 19 thermophilic endoglucanase protein sequences where the average percentage composition of the residues cysteine and histidine is only 0.28 and 0.74, which are less frequent in thermophilic proteins according to the statistics of amino acid composition based on MEGA 5 (Table 2).

**Table 2:** The frequencies of nineteen endoglucanases amino acids

	**Ala**	**Cys**	**Asp**	**Glu**	**Phe**	**Gly**	**His**	**Ile**	**Lys**	**Leu**	**Met**	**Asn**	**Pro**	**Gln**	**Arg**	**Ser**	**Thr**	**Val**	**Trp**	**Tyr**	**Total**
ADX81754	4.74	0.00	2.55	3.65	5.84	6.20	0.36	6.93	2.19	6.93	2.92	9.49	7.30	3.28	1.82	7.66	10.58	6.93	4.74	5.84	274.00
ACP37717	4.74	0.00	2.55	3.65	5.84	6.20	0.73	6.93	2.19	6.93	2.92	9.49	7.30	2.92	1.82	7.66	10.58	6.93	4.74	5.84	274.00
ADX84872	5.08	0.00	2.97	3.81	6.36	7.20	0.42	7.20	2.54	6.36	3.39	9.75	6.78	3.39	2.12	5.08	9.32	6.78	5.51	5.93	236.00
ACR41545	5.08	0.00	2.97	3.81	6.36	7.20	0.85	7.20	2.97	6.36	3.39	9.75	6.78	2.97	2.12	5.08	8.90	6.78	5.51	5.93	236.00
AAK42142	5.51	0.00	2.97	3.39	6.36	7.20	0.42	7.20	2.97	5.51	3.39	10.17	6.78	3.39	2.12	5.08	8.90	7.20	5.51	5.93	236.00
ADM27702	6.52	0.72	6.16	3.26	3.62	8.70	0.72	9.42	2.90	5.43	1.45	6.16	7.25	3.62	4.35	5.80	4.71	8.70	3.62	6.88	276.00
ADN02999	5.15	0.00	8.46	5.51	5.51	7.72	0.74	4.78	0.74	6.62	1.47	5.51	5.88	4.78	5.51	6.62	8.82	7.72	4.41	4.04	272.00
AEJ62362	5.15	0.00	8.09	6.25	5.51	7.72	0.74	4.04	0.74	6.62	1.10	5.51	5.88	4.41	5.51	6.62	8.82	8.82	4.41	4.04	272.00
AF181032	5.54	0.00	4.43	7.01	3.32	7.01	1.11	9.59	4.43	7.75	0.74	7.38	7.01	1.85	2.21	5.17	9.59	6.27	4.06	5.54	271.00
EEB73588	6.42	0.00	6.04	8.68	5.28	8.68	1.89	3.40	2.64	7.55	4.15	6.04	6.42	1.13	4.15	4.91	5.66	9.43	3.77	3.77	265.00
YP 003346783	3.97	0.40	6.35	7.94	7.14	6.75	1.19	4.37	6.75	5.56	1.98	5.95	4.76	1.98	1.59	4.76	7.94	10.32	4.76	5.56	252.00
Z69341	4.37	0.40	6.35	7.94	7.14	6.75	1.19	4.37	6.75	5.16	2.38	5.95	4.76	1.98	1.59	4.76	7.54	10.32	4.76	5.56	252.00
YP 002352530	5.43	0.00	4.26	8.53	4.26	5.04	1.16	9.69	9.30	5.81	1.94	7.75	4.65	1.55	2.33	5.43	5.04	6.59	4.26	6.98	258.00
AEA12777	10.71	0.40	4.37	6.35	4.76	7.54	0.00	5.16	3.57	5.95	3.97	3.17	7.14	2.78	3.57	8.33	4.76	6.75	4.37	6.35	252.00
AAY81158	2.90	0.36	5.07	2.54	5.80	7.97	0.72	8.33	2.90	7.97	2.90	9.78	3.99	3.26	1.45	7.61	7.97	8.70	2.17	7.61	276.00
AEB95090	2.89	0.00	4.33	4.33	5.42	7.94	0.36	6.14	3.25	8.66	4.33	7.22	5.42	2.89	2.17	10.11	5.78	7.58	2.53	8.66	277.00
ADN509821	3.90	1.42	2.48	3.90	3.19	7.09	0.71	9.22	3.19	9.22	3.19	11.35	6.74	1.42	1.77	7.80	4.61	6.03	4.61	8.16	282.00
ADL19785	3.96	0.00	3.24	3.60	3.24	12.59	0.36	6.12	0.72	11.51	4.32	7.19	5.76	2.16	2.88	7.91	5.76	8.63	3.96	6.12	278.00
CCC81038	9.13	1.66	4.98	4.56	3.32	9.13	0.41	2.49	1.24	9.96	1.24	3.32	7.47	2.49	6.22	7.88	4.15	9.96	3.32	7.05	241.00
Avg.	5.28	0.28	4.68	5.18	5.14	7.63	0.74	6.49	3.23	7.19	2.69	7.43	6.20	2.75	2.91	6.59	7.33	7.91	4.24	6.10	262.11

### Phylogenetic analysis

**Figure 1:**
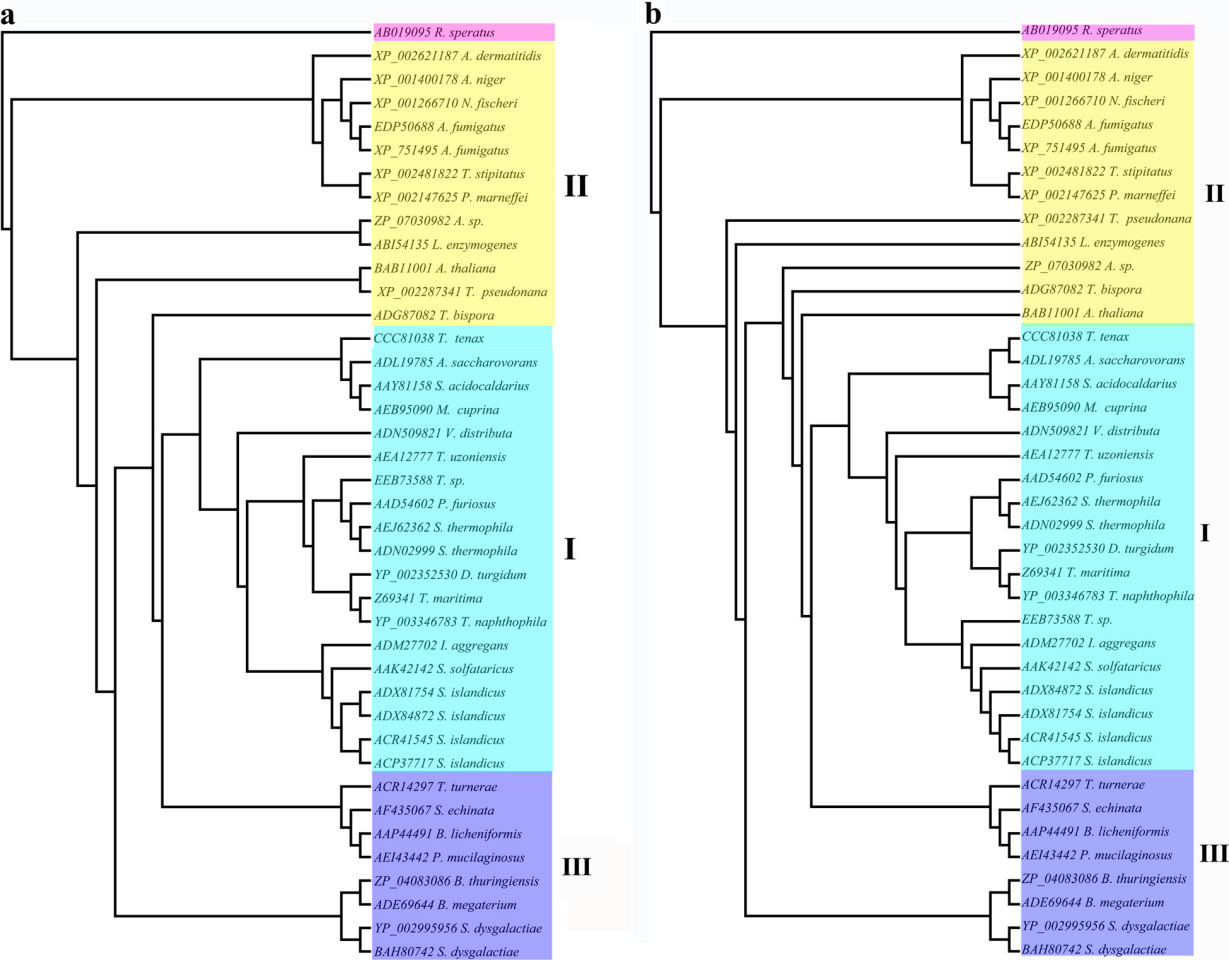
**The phylogenetic tree obtained using the endoglucanases and outgrouped by the protein sequence of *R. speratus*.** The NJ **(a)** and MP **(b)** tree were generated using program PAUP 4.0 beta 10 Win on 40 aligned amino acids. All the protein sequences are from Table 1. Proteins from hyperthermophilic bacteria and archaea are shown within light blue colored boxes **(I)**. Other proteins from bacteria, fungi and plants are shown within yellow **(II)** and blue **(III)** colored boxes.

**Figure 2:**
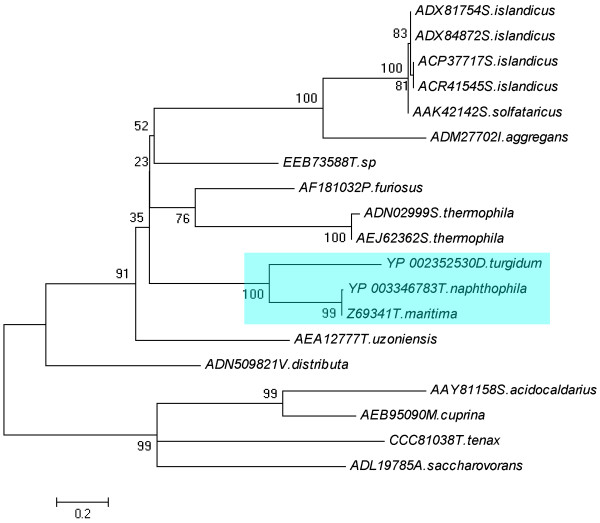
**The ML tree obtained using the 19 endoglucanases amino acids using program MEGA 5.** Numbers on nodes correspond to percentage bootstrap values for 1000 replicates.

Phylogenetic analysis based on the Maximum-parsimony (MP) and Neighbour-joining (NJ) procedure implemented in PAUP 4.0 [18] and other approaches (see Materials and Methods), indicated that all endoglucanase proteins can be reliably grouped into 3 distinct classes except for the outgroup *R. speratus*, which belongs to the insect family (Figure 1). Furthermore, from the multiple sequence alignments, the hyperthermophilic endoglucanase proteins belong to the class I, and others belong to class II and III. No obvious differentiations are implied in these 19 protein sequences. It was not surprising that there was a close relationship among 19 protein sequences from bacteria and archaea supported with good bootstrap values based on Maximum-likelihood (ML) tree by using MEGA 5 (Figure 2). It was inferred that the endoglucanases of *Dictyoglomus turgidum*, *Thermotoga naphthophila* and *Thermotoga maritima* which are currently studied in our research group are closely related compared to the others, although the identity of the amino acid sequences were shown less than 30% (Figure 1, Figure 2). Therefore, it was postulated that they may have a common origination based on protein evolution. Class II comprises of other 12 proteins from plant, fungi and bacteria, and class III comprises of 8 proteins from bacteria.

### Analysis of conserved and catalytic amino acid residues

**Figure 3:**
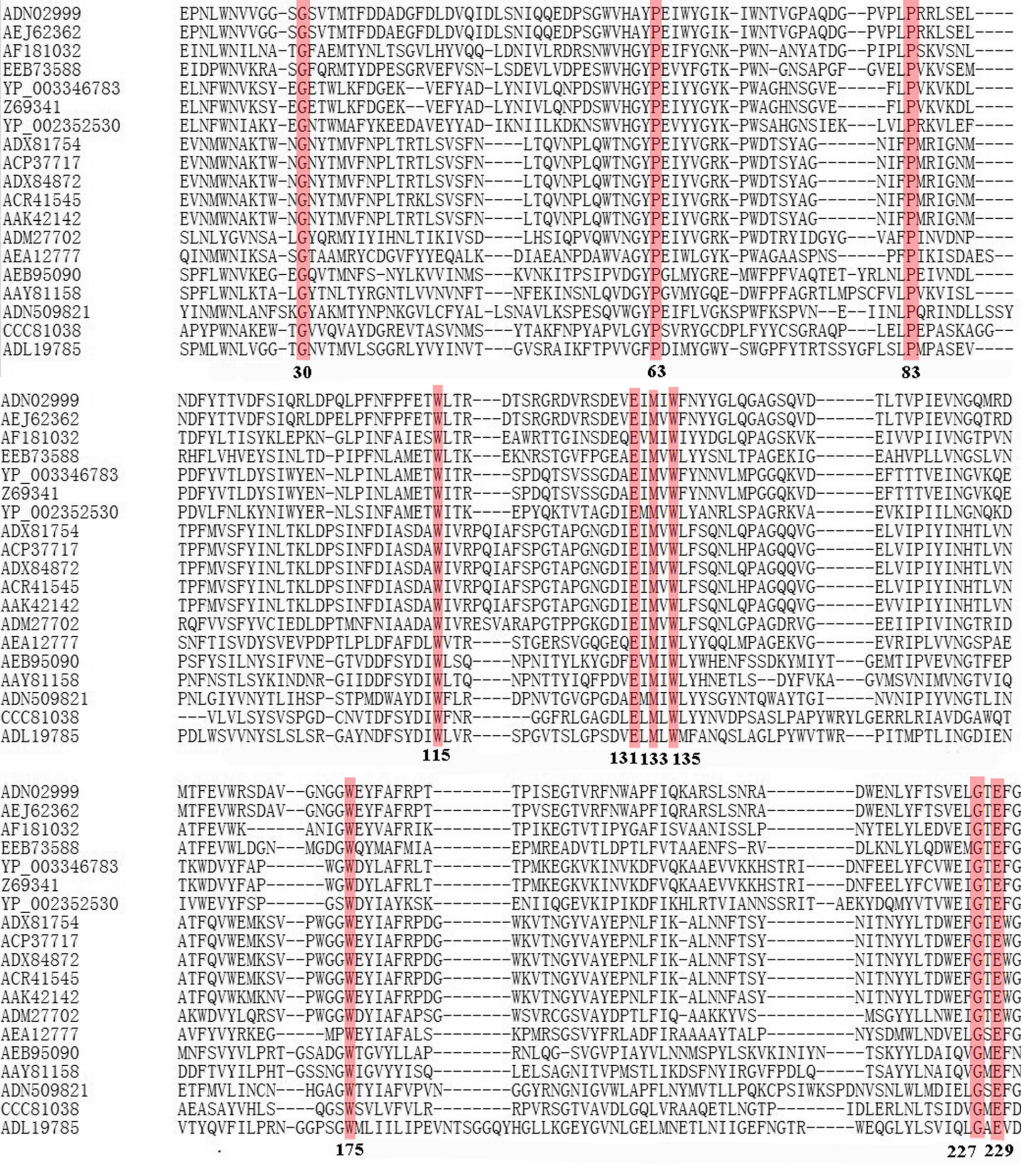
**Alignment of 19 endoglucanases amino acids sequences using CLUSTAL X2.0.** The highly conserved amino acids are colored in red.

For the further analysis of the relationship among 19 hyperthermophilic endoglucanases from bacteria and archaea, those 19 amino acid sequences were aligned again with Clustal X2 (Figure 3). We found that the conserved amino acids of hyperthermophilic endoglucanase in Cel12B (for instance) include Gly30, Pro63, Pro83, Trp115, Glu131, Met133, Trp135, Trp175, Gly227 and Glu229 which are highlighted in red (Figure 3), which is very different from the previously reported data [19,20]. Among these conserved amino acids, two glutamic acid residues might be the catalytic nucleophile and proton donor like lysozyme with acid base catalysis [21], other eight conserved amino acids might be necessary to the thermostability of protein and binding of the substrate.

### Hyperthermophilic protein homology modeling

**Figure 4:**
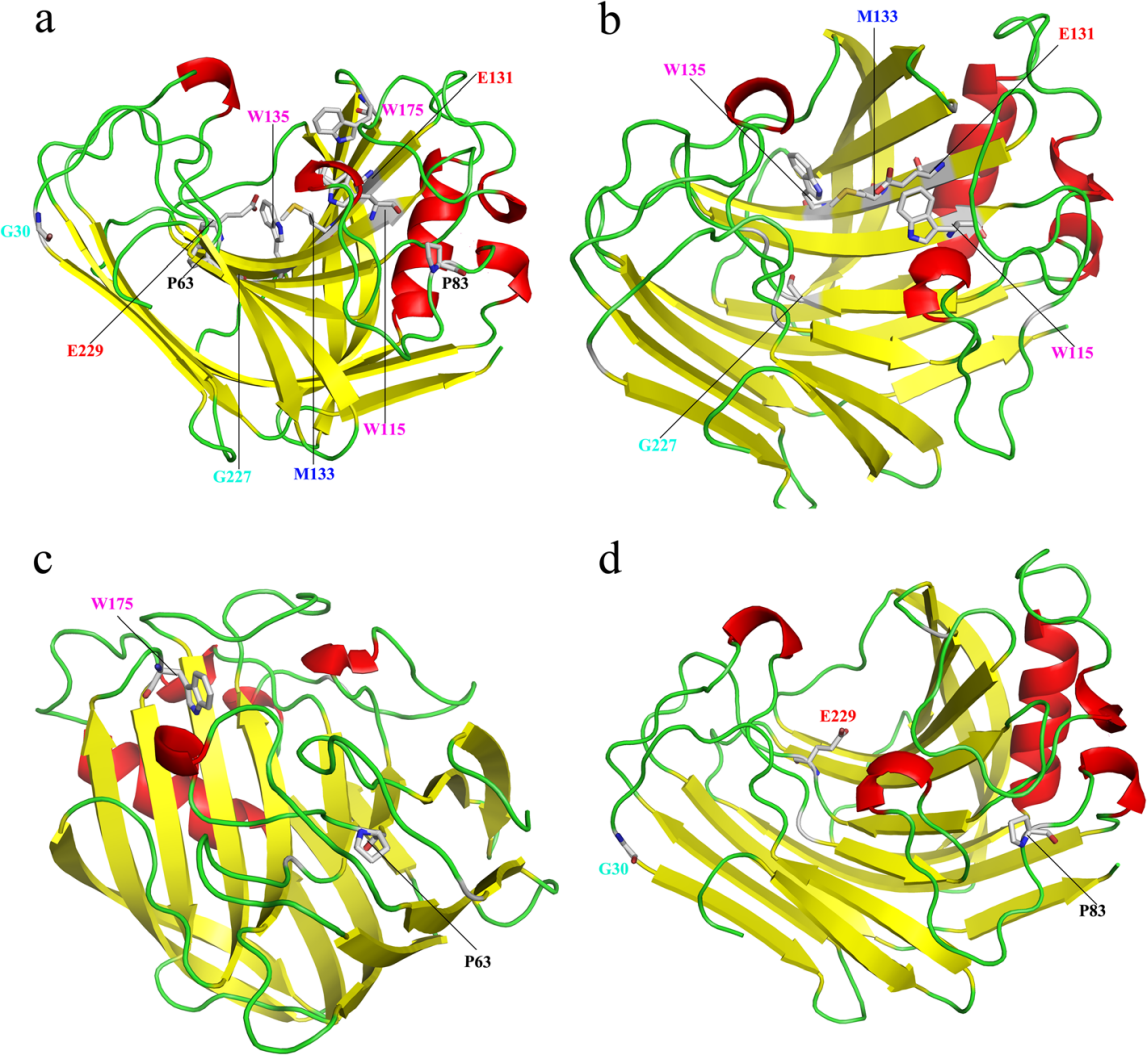
**Structure modeling of the protein Cel12B.** Different segments of the protein secondary structure are colored accordingly. The catalytic amino acids (Glu131 and Glu229) locating in the center of the structure were labeled in red **(a, b, d)**. The amino acids Trp115, Trp135 and Trp175 were labeled in magenta **(a, b, c)**, Met133 was labeled in blue **(a, b)**, where these four amino acids show a great importance in the substrate binding. The amino acids Pro63 and Pro83 were labeled in black **(a, c, d)**, Gly30 and Gly227 were labeled in cyan **(a, b, d)**, where these four amino acids are well related to the thermostability of the enzyme.

All the hyperthermophilic protein sequences were rendered using SWISS-MODEL database for protein modeling, but only one good model, Cel12B protein model from *T. maritima*, can be used to describe conserved amino acids in which sites of secondary structure and enzymatic center of protein. As described with Cel12B protein model, Glu131, Glu229, Trp115, Trp135, Trp175 and Met133 residues, comprised the active center of the protein (Figure 4a). Cel12B protein is primarily composed of β-sheet (Figure 4a,b,c,d). Trp115, Glu131, Met133, Trp135 and Gly227 residues are in the β-sheet; Pro63 and Trp175 residues are in the turn; and Gly30, Pro83 and Glu229 residues are in the random coil (Figure 4b,d).

### Analysis of site-directed mutagenesis

Base on the homology modeling, the functional amino acid residues Glu64, Pro63, Pro83 and Met133 of Cel12B were selected to be mutated. The results showed that the P63K, P83K, M133W, E64H, E64T and E64l mutant enzymes dramaticlly inhibited the enzyme activity of Cel12B toward CMC-Na, while E64S mutant protein apparently increased the enzyme activity (Table 3).

**Table 3:** Effect of site-directed mutagenesis on enzyme activity

**Strain**	**Optimum temp (°C)**	**Specific activity (U mg**^**-1**^**)**	**Relative activity (%)**
Control	90	105 ± 3.4	100 ± 3.2
E64T	85	53 ± 1.3	50 ± 1.2
E64H	85	25 ± 1.0	24 ± 1.0
E64L	ND	0	0
E64S	90	133 ± 2.5	127 ± 2.4
P63K	ND	0	0
P83K	ND	0	0
M133W	ND	0	0

*ND*: not determined. Values shown were the mean of triplicate experiments.

## Discussion

Endoglucanases isolated from hyperthermophilic organisms are more active and stable at higher temperatures than their counterparts from mesophiles. In addition, they may be more appropriate for degradation of the cellulose. Since the enzyme activity of those hyperthermophilic endoglucanases is not high for degradation, the hyperthermophilic modification by using genetic engineering is essential. Few structures on databases have been reported so far for transforming those enzymes. In this paper, nineteen sequences of hyperthermophilic endoglucanases were aligned and used for phylogenetic tree construction and molecular modeling to illustrate the relationship between structure and themostability.

The features of the nature environment of ancestral organism can be inferred by reconstructing phylogenetic tree using amino acid sequences of these organisms [22]. From the alignment of the amino acids sequences, the hyperthermophilic proteins from bacteria and archaea are clustered together based on the phylogenetic tree (Figure 1). Archaea, known to be an ancient organisms on earth, grow in strictly anaerobic environment (terrestrial solfataric springs, hydrothermal areas, and deep subsurface oil reservoirs) at high temperature (generally above 80°C), and hyperthermophilic bacteria also live in the same conditions [13,23]. Therefore, it is inferred that endoglucanases from hyperthermophilic microorganisms from GHF12 could share the similar enzymatic properties and catalytic mechanism.

The stability of thermophilic proteins depend on several amino acid residues and structural factors [24]. Specific amino acid composition plays a critical role in the thermostability of hyperthermophilic endoglucanase, with the fewest cysteine and histidine residues that are thermal stability among the whole protein sequences by using statistical comparison of the amino acid composition [25,26], Consistent with this feature, the average content of cysteine and histidine in our reserach is only 0.24 and 0.72 respectively (Table 2).

Ten conserved amino acids were found by the alignment of nineteen hyperthermophilic protein sequences (Figure 3), that we hypothesize may play a significant role in proton donation, substrate binding as well as the high thermostability. Among these nineteen amino acid sequences, only thethree-dimensional structure of endoglucanase from *T. maritima* could be obtained (Figure 4), since there is no suitable template for other proteins homologous modeling. Thus, the relationship between the ten amino acid residues of these endoglucanases and their molecular structures will be illustrated in Cel12B protein from *T. maritima*. The substitution of non-Gly residue with Gly residue can be used as one of the general strategies to enhance the protein stability [27,28]. In our study, residues Gly30 and Gly227 located in random coil and β-sheet, respectively, might contribute to the thermostability of the protein (Figure 4b,d).

It is believed that loop and turn are the weak connections among the protein secondary structure elements, but recently it was demonstrated that they played a key role in thermostability of protein, especially for the proteins that proline is located in loop or turn region [29]. Proline in the polypeptide chain possesses less conformational freedom than other amino acids, as the pyrrolidine ring of proline imposes rigid constrains on the N-C rotation and restricts the available conformational space of the preceding residue. Therefore it can bend the polypeptide chain on itself so as to prepare the backbone much more easily to form the hydrogen bonds with the polar side chains of other turns; meanwhile, the hydrophobic part of proline can interact with the adjacent hydrophobic cavity [30,31]. Compared to mesophilic proteins, thermophilic proteins contain more proline residues especially occurring at the turn, with higher frequency, as well as the shorter loop region of the glucosidase. As the consequence of the flexibility reduction of the polypeptide chain, the protein thermostability can be increased by introducing prolines at specific sites based on the facts that illustrated above [29,31,32]. Hence, residues Pro63 and Pro83, located in the turn and random coil respectively (Figure 4c,d), could provide closer packing of each region, as assumed for thermostability of protein. And then, it was finally confirmed by experimental results. Compared to other amino acids, lysine has longer side-chain groups and more vibrational degree of freedom, and it is more sensitive to the temperature. When the proline is substituted with lysine, the vibration of side-chain groups rises up at high temperature, and then the thermostability of the Cel12B decrease dramatically. Therefore, it is confirmed that residues Pro63 and Pro83 play an important role in stabilizing the Cel12B.

The crystal structure and protein molecular simulation supported that two glutamic acid residues are the catalytic nucleophile and proton donor that have been reported in many enzymes, lysozyme, xylanase as well as endoglucanase [33]. So, Glu131 (in β-sheet) and Glu229 (in random coil) residues are the proton donor and catalytic nucleophile repectively (Figure 4b,d). Although the chemical nature of the tryptophan residue in the catalytic center does not significantly affect the conformational properties of lysozyme, it exhibited a pronounced effect on the binding of substrate and the enhancement of the total enzyme activity [34]. It was reported that structural changes at the active site (W95L) of alcohol dehydrogenase from *Sulfolobus solfataricus* are consistent with the reduced activity on substrates and decreased coenzyme binding [35]. Therefore, we propose that three tryptophan residues (Trp115, 135 and 175, Figure 4b,c) of Cel12B protein may be essential in mediating the total cooperativity of the response of the enzyme to substrate. Met133, located in the middle of Trp135 and Glu131 in β-sheet (Figure 4b), is predicted to be related to the binding of substrate and also finally confirmed by experimental results. When it is replaced by tryptophan residue, the enzyme activity is significantly decreased. With the homology modeling result (data not shown), it is inferred that Glu64 is probably another functional acid amino located near the catalytic center. It is supposed that residue Glu64 might contribute to stabilizing the intermediate product. Maintaining the intermediate product may be caused by the interaction of side-chain group of Glu64. Polar amino acids, histidine and threonine are able to stabilize the intermediate product to some extent. However, their side-chain groups are relatively large, and possess larger steric hindrance, thus lead to decrease of the enzyme activity. Compared to glutamic acid, histidine and threonine, serine has smaller side-chain group and steric hindrance, so it can easily form hydrogen bond with product and stabilize it, and then increase the enzyme activity.

## Conclusions

Nineteen hyperthermophilic homologous protein sequences from GHF12 were aligned and used for constructing phylogenetic tree. It was inferred from the nodes that there is a close relationship among these nineteen homologous endoglucanases from hyperthermophilic bacteria and archaea. We have made clear the function of these conserved amino acids in Cel12B protein, which is helpful in analyzing other molecular structure and transforming them with site directed mutagenesis.

## Methods

### Extraction of sequences from databases

Thorough BLASTP searches for several divergent endoglucanases of plants, animals, bacteria, fungi, alga and archaea were performed to retrieve endoglucanases genes through NCBI, PDB (http://www.rcsb.org/pdb/home/home.do), UniProt (http://www.uniprot.org/) database server. Hyperthermophilic endoglucanase amino acid sequence was used (GenBank No: Z6934) [16] as a BLAST query for seeking hyperthermophilic endoglucanases from bacteria and archaea. New rounds of BLASTP searches for the nr protein and GenBank databases at NCBI restricted to plant or other organisms were carried out using representative endoglucanase from different classes of plants, bacteria, fungi and alga as queries.

### Multiple sequence alignment and phylogenetic analysis

One of the most widely used bioinformatics analysis is multiple sequences alignment, and it needs several widely used software packages to analysis. In this study, the multiple sequence alignment tool Clustal X2 was used for sequence alignment [36]. Sequences were further edited using the MEGA 5 when necessary and aligned manually [37]. In the phylogenetic analysis, sequences were trimmed so that only the relevant conserved domains were remained in the alignment. Phylogenetic relationships were inferred using the NJ and MP methods as implemented in PAUP 4.0 [18] while the Maximum-Likelihood method as implemented in MEGA 5 [37]. The NJ, MP and ML trees, displayed using TREEVIEW 1.6.6 (http://taxonomy.zoology.gla.ac.uk/rod/treeview.html), were evaluated with 1000 bootstrap replicates.

### Secondary structure prediction

For homology modeling, the crystal structure of the thermophilic endoglucanase (PDB ID: 3AAM) obtained from Protein Data Bank (PDB) was used as a template. The aligned sequences were submitted to SWISS-MODEL (http://www.expasy.org/swissmod/) to obtain the 3D structure of the endoglucanases [38-40]. The model was viewed using Swiss-PDB Viewer [41], and the quality of the model was evaluated by the local model quality estimation on SWISS-MODEL. The 3D structure of the protein was further modified by PyMOL (version 1.4.1, http://www.pymol.org/).

### Test of functional residues

Site-directed mutagenesis was used to analyze the related functional amino acid residues using reverse PCR. Restriction enzymes, DNA polymerase, *Dpn*I, T4 polynucleotide kinase and T4 ligase were purchased from Takara (Dalian, China) and used according to the manufacturer’s instructions. The sequence of *cel12B* gene (GenBank Protein No. Z69341) based on the *T. maritima* genomic DNA was amplified using primers 5′-GGAATTCCATATGAGGTGGGCAGTTCTTCTGA-3′, and 5′-CCGCTCGAGTTATTACTCGAGTTTTACACCTTCGACAGAGAAGTC-3′ (primers with the added compatible restriction sites of *Nde*I and *Xho*I, respectively). PCR was performed as follows: 94°C, 5 min; 30 cycles of 94°C for 30 s, 55°C for 30 s and 72°C for 50 s; and 72°C, 10 min. The recombinant vector was constructed as follows: the amplified PCR products were purified, digested with *Nde*I and *Xho*I, and then ligated into pET-20b vector at the corresponding sites. Reverse PCR amplifications were conducted by high-fidelity *Pyrobest* DNA polymerase using recombinant pET-20b-*cel12B* as templates, and primers were shown in Table 4. The templates were cleaned away from the products using *Dpn*I. Then, the resulting products were purified with BIOMIGA PCR Purification Kit (Shanghai, China), followed by phosphorylation using T4 polynucleotide kinase and finally ligated with T4 ligase. DNA sequencing was performed with ABI 3730 (Applied Biosystems, USA).

**Table 4:** Nucleotide sequences of used primers

**Primers**	**Nucleotide sequence**
Forward 1	5*'*-AGTAGAT**NNN**TGGATATCCATGCACCCAGC -3*'*
Reverse 1	5*'*-ACGGTTACAAGCCCTGGGCG -3*'*
Forward 2	5*'*- CATGGATAT**AAG**GAGATCTACTACGGTTACAAG -3′
Reverse 2	5*'*-CACCCAGCTGTCTGGATTCTGAAG -3*'*
Forward 3	5*'*-GAATTTCTT**AAG**CTGAAGGTGAAAGATCTTCC -3*'*
Reverse 3	5*'*-AACACCGCTGTTGTGCCCCG -3*'*
Forward 4	5*'*-CGGAGATC**TGG**GTTTGGTTCTACAACAACGTTC-3*'*
Reverse 4	5*'*-CGTCACCCGAAGAAACAGAGGTC -3*'*

*E. coli* BL21 (DE3) cells harboring recombinants were grown at 37°C and 200 rpm in 200 mL of Luria-Bertani (LB) with appropriate antibiotic selection. When the OD_600_ reached 0.6-0.8, the expression of mutated enzymes were induced by the addition of 0.5 mM isopropyl β-D-1-thiogalactopyranoside (IPTG) and the culture was incubated at 37°C and 200 rpm for 5 h. Cells were harvested by centrifugation at 4°C (10000 rpm, 5 min), washed twice with 20 mM Tris-HCl buffer (pH 8.0), and re-suspended in 5 mL of 5 mM imidazole, 0.5 M NaCl, and 20 mM Tris-HCl buffer (pH 7.9). All subsequent steps were carried out at 4°C. The cell extracts after sonication were heat treated at 50°C for 30 min, cooled in an ice bath, and then centrifuged (15000 g, 4°C, 20 min). The resulting supernatants were loaded onto a 1 ml Ni^2+^ affinity column (Novagen, USA) and the bounded proteins were eluted by discontinuous imidazole gradient.

Enzyme activity was determined using 5-dinitrosalicylic acid (DNS) method [42]. The reaction mixture, containing 50 mM imidazole-potassium buffer (pH 6.0), 0.5% sodium carboxymethyl cellulose (CMC-Na), and a certain amount of endoglucanase (0.1 μg) in 0.2 mL, was incubated for 10 min at 85°C. The reaction was stopped by the addition of 0.3 mL DNS. The absorbance of the mixture was measured at 520 nm. One unit of enzyme activity was defined as the amount of enzyme necessary to liberate 1 μmol of reducing sugars per min under the assay conditions. All the values of enzymatic activities shown in figures were averaged from three replicates.

## Competing interests

The authors declare that they have no competing interests.

## Authors’ contributions

HS conceived the project, carried out phylogenetic phylogenetic analysis, LW, XL, YZ and WL carried out database searches and protein modeling, and FW and XL supervised the work. HS, XL, and FW wrote the manuscript. All authors read and approved the final manuscript.

## References

[B1] Wang T Liu X Yu Q Zhang X Qu Y Gao P Directed evolution for engineering pH profile of endoglucanase III from *Trichoderma reesei* Biomol Eng 2005 22 1–3 89 94 15857788 10.1016/j.bioeng.2004.10.003

[B2] Liang C Fioroni M Rodriguez-Ropero F Xue Y Schwaneberg U Ma Y Directed evolution of a thermophilic endoglucanase (Cel5A) into highly active Cel5A variants with an expanded temperature profile J Biotechnol 2011 154 1 46 53 10.1016/j.jbiotec.2011.03.025 21501637

[B3] Anbar M Lamed R Bayer EA Thermostability enhancement of *Clostridium thermocellum* cellulosomal endoglucanase Cel8A by a single glycine substitution Chemcatchem 2010 2 8 997 1003 10.1002/cctc.201000112

[B4] Nakazawa H Okada K Onodera T Ogasawara W Okada H Morikawa Y Directed evolution of endoglucanase III (Cel12A) from trichoderma reesei Appl Microbiol Biotechnol 2009 83 4 649 657 10.1007/s00253-009-1901-3 19205687

[B5] Watanabe H Noda H Tokuda G Lo N A cellulase gene of termite origin Nature 1998 394 6691 330 331 10.1038/28527 9690469

[B6] Davison A Ancient origin of glycosyl hydrolase family 9 cellulase genes Mol Biol Evol 2005 22 5 1273 1284 10.1093/molbev/msi107 15703240

[B7] Mardanov AV Svetlitchnyi VA Beletsky AV Prokofeva MI Bonch-Osmolovskaya EA Ravin NV Skryabin KG The genome sequence of the crenarchaeon *Acidilobus saccharovorans* supports a new order, *Acidilobales*, and suggests an important ecological role in terrestrial acidic hot Springs Appl Environ Microbiol 2010 76 16 5652 5657 10.1128/AEM.00599-10 20581186 PMC2918975

[B8] Reno ML Held NL Fields CJ Burke PV Whitaker RJ Biogeography of the *Sulfolobus islandicus* pan-genome Proc Natl Acad Sci 2009 106 21 8605 8610 10.1073/pnas.0808945106 19435847 PMC2689034

[B9] Guo L Brugger K Liu C Shah SA Zheng H Zhu Y Wang S Lillestol RK Chen L Frank J Genome analyses of Icelandic strains of *Sulfolobus islandicus*, model organisms for genetic and virus-host interaction studies J Bacteriol 2011 193 7 1672 1680 10.1128/JB.01487-10 21278296 PMC3067641

[B10] Göker M Held B Lapidus A Nolan M Spring S Yasawong M Lucas S Glavina Del Rio T Tice H Cheng J-F Complete genome sequence of *Ignisphaera aggregans* type strain (AQ1.S1T) Stand Genomic Sci 2010 3 1 66 75 10.4056/sigs.1072907 21304693 PMC3035270

[B11] Angelov A Liebl S Ballschmiter M Boemeke M Lehmann R Liesegang H Daniel R Liebl W Genome sequence of the polysaccharide-degrading, thermophilic anaerobe *Spirochaeta thermophila* DSM 6192 J Bacteriol 2010 192 24 6492 6493 10.1128/JB.01023-10 20935097 PMC3008529

[B12] Mardanov AV Gumerov VM Beletsky AV Prokofeva MI Bonch-Osmolovskaya EA Ravin NV Skryabin KG Complete genome sequence of the thermoacidophilic crenarchaeon *Thermoproteus uzoniensis* 768*–*20 J Bacteriol 2011 193 12 3156 3157 10.1128/JB.00409-11 21478349 PMC3133184

[B13] Chen LM Brugger K Skovgaard M Redder P She QX Torarinsson E Greve B Awayez M Zibat A Klenk HP The genome of *Sulfolobus acidocaldarius*, a model organism of the *Crenarchaeota* J Bacteriol 2005 187 14 4992 4999 10.1128/JB.187.14.4992-4999.2005 15995215 PMC1169522

[B14] Liu L-J You X-Y Zheng H Wang S Jiang C-Y Liu S-J Complete genome sequence of *Metallosphaera cuprina*, a metal sulfide-oxidizing archaeon from a hot spring J Bacteriol 2011 193 13 3387 3388 10.1128/JB.05038-11 21551305 PMC3133273

[B15] Wu D Hugenholtz P Mavromatis K Pukall R Dalin E Ivanova NN Kunin V Goodwin L Wu M Tindall BJ A phylogeny-driven genomic encyclopaedia of bacteria and archaea Nature 2009 462 7276 1056 1060 10.1038/nature08656 20033048 PMC3073058

[B16] Liebl W Ruile P Bronnenmeier K Riedel K Lottspeich F Greif I Analysis of a *Thermotoga maritima* DNA fragment encoding two similar thermostable cellulases, CelA and CelB, and characterization of the recombinant enzymes Microbiol (Reading, England) 1996 142 Pt 9 2533 2542 10.1099/00221287-142-9-2533 8828221

[B17] Zhaxybayeva O Swithers KS Lapierre P Fournier GP Bickhart DM DeBoy RT Nelson KE Nesbo CL Doolittle WF Gogarten JP On the chimeric nature, thermophilic origin, and phylogenetic placement of the thermotogales Proc Natl Acad Sci U S A 2009 106 14 5865 5870 10.1073/pnas.0901260106 19307556 PMC2667022

[B18] Wilgenbusch JC Swofford D Inferring evolutionary trees with PAUP Curr Protoc Bioinformatics 2003 Chaper 6, unit 6.4. http://www.currentprotocols.com/protocol/bi0604 10.1002/0471250953.bi0604s00 18428704

[B19] Chhabra SR Shockley KR Ward DE Kelly RM Regulation of endo-acting glycosyl hydrolases in the hyperthermophilic bacterium *Thermotoga maritima* grown on glucan- and mannan-based polysaccharides Appl Environ Microbiol 2002 68 2 545 554 10.1128/AEM.68.2.545-554.2002 11823189 PMC126696

[B20] Wang Y Wang X Tang R Yu S Zheng B Feng Y A novel thermostable cellulase from *Fervidobacterium nodosum* J Mol Catal B Enzym 2010 66 3–4 294 301

[B21] Sinnott ML Catalyic mechanisms of enzymatic glycosyl transfer Chem Rev 1990 90 7 1171 1202 10.1021/cr00105a006

[B22] Gaucher EA Thomson JM Burgan MF Benner SA Inferring the palaeoenvironment of ancient bacteria on the basis of resurrected proteins Nature 2003 425 6955 285 288 10.1038/nature01977 13679914

[B23] Mardanov AV Ravin NV Svetlitchnyi VA Beletsky AV Miroshnichenko ML Bonch-Osmolovskaya EA Skryabin KG Metabolic versatility and Indigenous origin of the archaeon *Thermococcus sibiricus*, isolated from a siberian oil reservoir, as revealed by genome analysis Appl Environ Microbiol 2009 75 13 4580 4588 10.1128/AEM.00718-09 19447963 PMC2704819

[B24] Kumar S Tsai CJ Nussinov R Factors enhancing protein thermostability Protein Eng 2000 13 3 179 191 10.1093/protein/13.3.179 10775659

[B25] Warren GL Petsko GA Composition analysis of alpha-helices in thermophilic organisms Protein Eng 1995 8 9 905 913 10.1093/protein/8.9.905 8746728

[B26] Kumar S Bansal M Dissecting alpha-helices: position-specific analysis of alpha-helices in globular proteins Proteins 1998 31 4 460 476 10.1002/(SICI)1097-0134(19980601)31:4<460::AID-PROT12>3.0.CO;2-D 9626705

[B27] Kimura S Kanaya S Nakamura H Thermostabilization of *Escherichia coli* ribonuclease HI by replacing left-handed helical Lys95 with Gly or Asn J Biol Chem 1992 267 31 22014 22017 1331044

[B28] Kawamura S Kakuta Y Tanaka I Hikichi K Kuhara S Yamasaki N Kimura M Glycine-15 in the bend between two alpha-helices can explain the thermostability of DNA binding protein HU from *Bacillus stearothermophilus* Biochemistry 1996 35 4 1195 1200 10.1021/bi951581l 8573574

[B29] Watanabe K Kitamura K Suzuki Y Analysis of the critical sites for protein thermostabilization by proline substitution in oligo-1,6-glucosidase from *Bacillus coagulans* ATCC 7050 and the evolutionary consideration of proline residues Appl Environ Microbiol 1996 62 6 2066 2073 8787404 10.1128/aem.62.6.2066-2073.1996 PMC167984

[B30] Suzuki Y Oishi K Nakano H Nagayama T A strong correlation between the increase in mumber of proline resdues and the rise in thermostability of 5 *Bacillus* oligo-1,6-glucsidases Appl Microbiol Biotechnol 1987 26 6 546 551 10.1007/BF00253030

[B31] Zhu GP Xu C Teng MK Tao LM Zhu XY Wu CJ Hang J Niu LW Wang YZ Increasing the thermostability of D-xylose isomerase by introduction of a proline into the turn of a random coil Protein Eng 1999 12 8 635 638 10.1093/protein/12.8.635 10469823

[B32] Suzuki Y A general principle of increasing protein thermostability Proc Japan Acad Series B-Physl and Bio Sci 1989 65 6 146 148 10.2183/pjab.65.146

[B33] Derewenda U Swenson L Green R Wei Y Morosoli R Shareck F Kluepfel D Derewenda ZS Crystal structure, at 2.6-A resolution, of the *streptomyces lividans* xylanase a, a member of the F family of beta-1,4-D-glycanases J bio chem 1994 269 33 20811 20814 8063693

[B34] Churakova NI Cherkasov IA Kravchenko NA The role of the tryptophan-62 residue in the structure and function of lysozyme Biokhimii͡a (Moscow, Russia) 1977 42 2 274 276 15655

[B35] Pennacchio A Esposito L Zagari A Rossi M Raia CA Role of Tryptophan 95 in substrate specificity and structural stability of *Sulfolobus solfataricus* alcohol dehydrogenase Extremophiles 2009 13 5 751 761 10.1007/s00792-009-0256-0 19588068

[B36] Larkin MA Blackshields G Brown NP Chenna R McGettigan PA McWilliam H Valentin F Wallace IM Wilm A Lopez R Clustal W and clustal X version 2.0 Bioinformatics 2007 23 21 2947 2948 10.1093/bioinformatics/btm404 17846036

[B37] Tamura K Peterson D Peterson N Stecher G Nei M Kumar S MEGA5: Molecular evolutionary genetics analysis using maximum likelihood, evolutionary distance, and maximum parsimony methods Mol Biol Evol 2011 28 10 2731 2739 10.1093/molbev/msr121 21546353 PMC3203626

[B38] Schwede T Kopp J Guex N Peitsch MC SWISS-MODEL: an automated protein homology-modeling server Nucleic Acids Res 2003 31 13 3381 3385 10.1093/nar/gkg520 12824332 PMC168927

[B39] Guex N Peitsch MC SWISS-MODEL and the Swiss-PdbViewer: an environment for comparative protein modeling Electrophoresis 1997 18 15 2714 2723 10.1002/elps.1150181505 9504803

[B40] Arnold K Bordoli L Kopp J Schwede T The SWISS-MODEL workspace: a web-based environment for protein structure homology modelling Bioinformatics 2006 22 2 195 201 10.1093/bioinformatics/bti770 16301204

[B41] Kaplan W Littlejohn TG Swiss-PDB viewer (deep view) Brief Bioinform 2001 2 2 195 197 10.1093/bib/2.2.195 11465736

[B42] Miller GL Use of dinitrosalicylic acid reagent for determination of ruducing sugar Anal Chem 1959 31 3 426 428 10.1021/ac60147a030

